# The relevance of qualitative research in nursing: exploring experiences and context in care

**DOI:** 10.1590/0034-7167.202578supl301

**Published:** 2025-08-08

**Authors:** Ellen Synthia de Oliveira, Maria Helena Presado, Cristina Lavareda Baixinho

**Affiliations:** I Universidade Federal de Góias, Graduate Program in Public Health. Goiânia, Góias, Brazil; II Escola Superior de Enfermagem de Lisboa, Centro de Investigação, Inovação e Desenvolvimento em Enfermagem de Lisboa. Lisbon, Portugal; III Center for Innovative Care and Health Technology (ciTechcare). Leiria, Portugal

Healthcare systems face complex challenges. Population aging, the increasing prevalence of chronic diseases, and dependence on self-care are all factors that contribute to the changing profile of healthcare clients and their needs. These factors are also among the main causes of rising healthcare costs.

The public debate surrounding these issues highlights the need for policies that promote self-care, integrated care, and a personand family-centered care model. Such policies aim to ensure adherence to therapies and rehabilitation programs, with an emphasis on behavioral change toward more active and participatory lifestyles, as well as empowering citizens, families, and communities to promote self-care and well-being^(1)^.

These guidelines also represent a challenge for health researchers, especially nurses, who must develop studies that deepen the understanding of people’s experiences in their health-illness transition processes. This includes exploring their difficulties, needs and decision-making in relation to the pharmacological and non-pharmacological therapies prescribed to them.

Research that is closer to citizens, taking into account their values, preferences and motivations, has the potential to transform healthcare. It contributes to access to clinical practice environments, especially for vulnerable populations, promoting person-centered care and empowering individuals in their autonomy and responsibility in the health-disease process, within the context of their life projects^(2)^.

In this regard, interpretative or mixed-methods studies, due to their qualitative nature, can capture these specificities that emerge from experiences, behaviors and choices. These studies reinforce the World Health Organization recommendations, which advocates that self-care and interventions to promote it are essential components for achieving universal health coverage, promoting health, keeping the world safe and serving vulnerable populations^(1)^.

Qualitative research is also essential for the sustainability of healthcare systems. It recognizes that qualitative methods are fundamental for implementing knowledge, supporting evidence-based practice and adapting global knowledge to the specific sociocultural, resource and characteristics of local healthcare systems^(3)^.

This editorial aims to invite researchers to rethink the design of their studies, seeking a deeper understanding of experiences and contexts. Moreover, it proposes a reflection on the models used to ensure person-centered care that promotes self-care, respects cultural specificities and considers the resources available in different areas of knowledge.
