# Supplementary material for: The relevance of qualitative research in nursing: exploring experiences and context in care
Source: Rev Bras Enferm. 2025 Aug 8;78(Suppl 3):e78supl301. doi: 10.1590/0034-7167.202578supl301 (PMC12334126; doi:10.1590/0034-7167.202578supl301)
Supplement: Translation

# Translation: A relevância da pesquisa qualitativa na enfermagem: explorando vivências e contexto no cuidado

**DOI:** 10.1590/0034-7167.202578supl301pt

**Authors:** Ellen Synthia de Oliveira, Maria Helena Presado, Cristina Lavareda Baixinho

**Affiliations:** I Universidade Federal de Góias, Programa de Pós-Graduação em Saúde Coletiva. Goiânia, Góias, Brasil; II Escola Superior de Enfermagem de Lisboa, Centro de Investigação, Inovação e Desenvolvimento em Enfermagem de Lisboa. Lisboa, Portugal; III Center for Innovative Care and Health Technology (ciTechcare). Leiria, Portugal

Os sistemas de saúde enfrentam desafios complexos. O envelhecimento populacional, o aumento da prevalência de doenças crônicas e a dependência para o autocuidado são fatores que contribuem para a alteração do perfil dos clientes dos cuidados de saúde e de suas necessidades. Esses fatores também estão entre as principais causas do aumento dos custos com a saúde.

O debate público em torno dessas questões destaca a necessidade de políticas que promovam o autocuidado, a integração dos cuidados e um modelo de cuidado centrado na pessoa e em sua família. Tais políticas visam garantir a adesão às terapêuticas e aos programas de reabilitação, com ênfase na mudança comportamental para estilos de vida mais ativos e participativos, além da capacitação de cidadãos, famílias e comunidades para a promoção do autocuidado e bem-estar^(1)^.

Essas orientações também representam um desafio para os investigadores na área da saúde, especialmente enfermeiros, que devem desenvolver estudos que aprofundem a compreensão das vivências das pessoas em seus processos de transição saúde-doença. Isso inclui explorar suas dificuldades, necessidades e tomadas de decisão em relação às terapias farmacológicas e não farmacológicas que lhes são prescritas.

Uma investigação mais próxima dos cidadãos, que leve em conta seus valores, preferências e motivações, tem o potencial de transformar os cuidados de saúde. Ela contribui para o acesso aos ambientes de prática clínica, especialmente para populações vulneráveis, promovendo um cuidado centrado na pessoa e empoderando os indivíduos em sua autonomia e responsabilidade no processo de saúde-doença, dentro do contexto de seus projetos de vida^(2)^.

Nesse sentido, estudos interpretativistas ou de métodos mistos, por sua natureza qualitativa, podem captar essas especificidades que emergem das vivências, experiências, comportamentos e escolhas. Esses estudos reforçam as recomendações da Organização Mundial da Saúde, que defende que o autocuidado e as intervenções para sua promoção sejam componentes essenciais para alcançar a cobertura universal de saúde, promovendo a saúde, mantendo o mundo seguro e atendendo às populações vulneráveis^(1)^.

A pesquisa qualitativa também é essencial para a sustentabilidade dos sistemas de saúde. Ela reconhece que os métodos qualitativos são fundamentais para a implementação de conhecimento, apoiando a prática baseada em evidências e a adaptação do conhecimento global às especificidades socioculturais, recursos e características dos sistemas de saúde locais^(3)^.

Este editorial tem como objetivo convidar os investigadores a repensarem o desenho de seus estudos, buscando uma compreensão mais profunda das vivências e dos contextos. Além disso, propõe uma reflexão sobre os modelos utilizados para garantir um cuidado centrado na pessoa que promova o autocuidado, respeite as especificidades culturais e considere os recursos disponíveis em diferentes áreas do conhecimento.

# Translation: La relevancia de la investigación cualitativa en enfermería: explorando experiencias y contexto en el cuidado

**DOI:** 10.1590/0034-7167.202578supl301es

**Authors:** Ellen Synthia de Oliveira, Maria Helena Presado, Cristina Lavareda Baixinho

**Affiliations:** I Universidade Federal de Góias, Programa de Pós-Graduação em Saúde Coletiva. Goiânia, Góias, Brasil; II Escola Superior de Enfermagem de Lisboa, Centro de Investigação, Inovação e Desenvolvimento em Enfermagem de Lisboa. Lisboa, Portugal; III Center for Innovative Care and Health Technology (ciTechcare). Leiria, Portugal

Los sistemas de salud se enfrentan a desafíos complejos. El envejecimiento poblacional, la creciente prevalencia de enfermedades crónicas y la dependencia del autocuidado son factores que contribuyen al cambio en el perfil de los usuarios de la atención médica y sus necesidades. Estos factores también se encuentran entre las principales causas del aumento de los costos de la atención médica.

El debate público en torno a estos temas resalta la necesidad de políticas que promuevan el autocuidado, la atención integral y un modelo de atención centrado en la persona y la familia. Dichas políticas buscan garantizar la adherencia a las terapias y programas de rehabilitación, con énfasis en el cambio de comportamiento hacia estilos de vida más activos y participativos, así como empoderar a la ciudadanía, las familias y las comunidades para promover el autocuidado y el bienestar^(1)^.

Estas directrices también representan un reto para los investigadores en salud, especialmente el personal de enfermería, quienes deben desarrollar estudios que profundicen en la comprensión de las experiencias de las personas en sus procesos de transición salud-enfermedad. Esto incluye explorar sus dificultades, necesidades y toma de decisiones en relación con las terapias farmacológicas y no farmacológicas que se les prescriben.

Una investigación más cercana a la ciudadanía, que tenga en cuenta sus valores, preferencias y motivaciones, tiene el potencial de transformar la atención sanitaria. Contribuye al acceso a entornos de práctica clínica, especialmente para poblaciones vulnerables, promoviendo una atención centrada en la persona y empoderando a las personas en su autonomía y responsabilidad en el proceso salud-enfermedad, en el contexto de sus proyectos de vida^(2)^.

En este sentido, los estudios interpretativos o de métodos mixtos, debido a su naturaleza cualitativa, pueden captar estas especificidades que surgen de las experiencias, los comportamientos y las elecciones. Estos estudios refuerzan las recomendaciones de la Organización Mundial de la Salud, que defiende que el autocuidado y las intervenciones para promoverlo son componentes esenciales para lograr la cobertura sanitaria universal, promover la salud, mantener un mundo seguro y atender a las poblaciones vulnerables^(1)^.

La investigación cualitativa también es esencial para la sostenibilidad de los sistemas de salud. Reconoce que los métodos cualitativos son fundamentales para implementar el conocimiento, respaldar la práctica basada en la evidencia y adaptar el conocimiento global a las características socioculturales, de recursos y específicas de los sistemas de salud locales^(3)^.

Este editorial busca invitar a los investigadores a repensar el diseño de sus estudios, buscando una comprensión más profunda de las experiencias y los contextos. Además, propone una reflexión sobre los modelos utilizados para garantizar una atención centrada en la persona que promueva el autocuidado, respete las especificidades culturales y considere los recursos disponibles en diferentes áreas del conocimiento.
